# High inbreeding and low connectivity among *Ambystoma texanum* populations in fragmented Ohio forests

**DOI:** 10.1002/ece3.3637

**Published:** 2017-11-15

**Authors:** Elizabeth A. Rhoads, Patrick Kelly Williams, Carissa M. Krane

**Affiliations:** ^1^ Department of Biology University of Dayton Dayton OH USA

**Keywords:** *Ambystoma texanum*, habitat fragmentation, landscape genetics, microsatellites, mole salamanders, population structure

## Abstract

Habitat loss and fragmentation negatively impact the size and diversity of many natural populations. Woodland amphibians require connected aquatic and terrestrial habitats to complete their life cycle, and often rely on metapopulation structure for long‐term persistence. Wetland loss and deforestation fragment amphibian populations, which may result in population isolation and its negative effects. The aim of this research was to analyze the population genetic structure of small‐mouthed salamanders (*Ambystoma texanum*) in western Ohio, where agriculture is now the dominant land use. Salamander tail tissue was collected from eight breeding pools. Three pools occur in the same forest; the other five are in forest patches at distances ranging from 250 m to 20 km from one another. Eight microsatellite loci were amplified by PCR and genotyped for allele size. Observed heterozygosities were lower than expected in all sampled populations; the two most isolated sites (Ha1, Ha2) had the highest inbreeding coefficients. Ha2 also had the lowest mean number of alleles and was found to be genetically differentiated from populations to which our data analysis indicates it was historically connected by gene flow. The most distant site (Ha1) had the highest number of private alleles and showed genetic differentiation from other populations both historically and currently. Geographic distance between pools was strongly correlated with the number of private alleles in a population. The results suggest that population isolation results in decreased genetic diversity and that a breakdown of metapopulation structure due to landscape change may contribute to differentiation between once‐connected populations.

## 1 INTRODUCTION

Habitat destruction and fragmentation are the leading cause of species declines and extinctions worldwide (Fahrig, 2003). Habitat patches may contain small populations that are isolated from conspecifics in patches that are too far away or unreachable due to migration barriers. Isolated populations are at great risk of extinction from either stochastic or deterministic causes, as their genetic diversity and biological fitness are reduced over time (Allentoft & O'Brien, 2010).

The eastern North American landscape has been significantly altered in the last two hundred years, mainly through deforestation. In western Ohio today, small forest patches are common in an agricultural landscape (Gordon, 1969). These patches often contain vernal pools that serve as breeding habitat for woodland amphibians. Loss of both terrestrial and aquatic habitats for amphibians has occurred, as wetland drainage accompanied forest clearing for agriculture (Dahl, 1990). Many amphibian species in this region are in decline (Lannoo, 1998), and spatial isolation due to fragmentation may be a serious threat to their survival as woodland amphibians prefer not to traverse nonforested landscapes (Pittman & Semlitsch, 2013; Rothermel, 2004; Rothermel & Semlitsch, 2002).

Amphibian extinctions are occurring at an alarming rate around the globe (Blaustein, Wake, & Sousa, 1994; McCallum, 2007), and the principal driver of extinction is habitat change (Brooks et al., 2002). Many amphibian species have metapopulation structure that requires habitat connectivity between geographically close breeding populations (Marsh & Trenham, 2001). Low vagility, and sensitivity to migration barriers produced by habitat fragmentation and other anthropogenic development, limits migration between breeding populations (Semlitsch & Bodie, 2003). Amphibian species conservation must utilize molecular tools to understand the genetic impact of habitat change (McCartney‐Melstad & Shaffer, 2015).

Identifying the geographic scale at which gene flow is interrupted by habitat fragmentation is crucial for prescribing conservation and management plans for amphibians. Scale can vary widely depending on the species’ sensitivity, migratory ability, and the effective population sizes of isolated populations (Jacobs & Houlahan, 2011; Richardson, 2012; Scott, Komoroski, Croshaw, & Dixon, 2013). A consensus of landscape genetics studies suggests that a species‐specific approach is necessary for assessing the effect of habitat change on population genetics (Storfer, Murphy, Spear, Holderegger, & Waits, 2010). Factors including geographic distance between breeding pools, intervening land use, and occurrence of migration barriers are important in influencing the connectivity among populations of amphibians at both a landscape and fine scale, and may impact the genetic diversity of each population.

Mole salamanders (*Ambystoma*) inhabit terrestrial areas that contain ephemeral breeding pools, to which adults exhibit high fidelity (Shoop, 1968; Shoop & Doty, 1972). Forest fragmentation may disrupt migration between breeding populations due to their low vagility. A study of the spotted salamander (*A. maculatum*) in northeastern Ohio found that gene flow may occur in a highly fragmented landscape, possibly along riparian zones (Purrenhage, Niewiarowski, & Moore, 2009), while others have found genetic differentiation among breeding populations in the same forest patch (Bartoszek, 2009) and across a small migration barrier such as a railroad track (Bartoszek & Greenwald, 2009).

The aim of this study was to investigate the impact of forest fragmentation on the genetic diversity and population structure of the small‐mouthed salamander (*Ambystoma texanum*) at both the landscape and fine spatial scales in western Ohio. This species’ range extends throughout most of eastern North America, where it resides in forests that contain vernal pools (Petranka, 1998). Small‐mouthed salamanders may disperse up to 125 m from their natal pool (Williams, 1973). This species is one of the smallest Ambystomids (Petranka, 1998), and its dispersal ability may be more limited than larger species (Semlitsch & Bodie, 2003).

**Figure 1:**
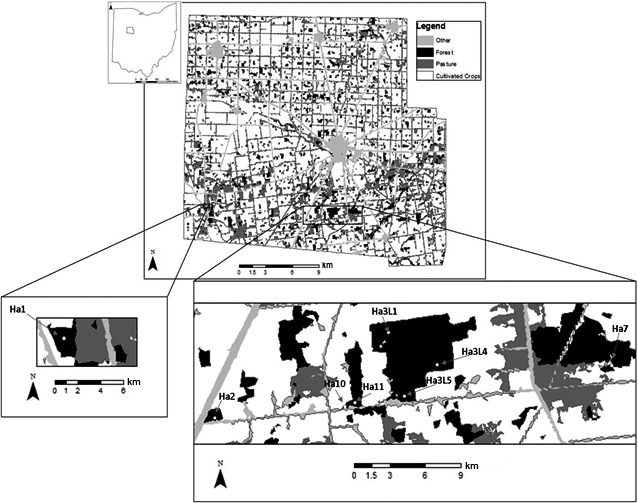
Map of sampled populations in Hardin County, Ohio,USA

Given the highly fragmented nature of forest patches in western Ohio, the main concern is that a lack of connectivity between breeding populations in separate forest patches eliminates metapopulation structure, results in reduced genetic diversity, and may lead to local extinctions. In the mid‐1800s, continuous forest in Hardin County, Ohio, was cleared for farming. Since settlement, the county is heavily agricultural (USDA 2009), yet contains multiple forest patches with vernal pools (Figure 1). The spatial arrangement of the forest patches in the study area provided an opportunity to approach this question from both a landscape and fine scale. Prior studies have shown the importance of both of these scales for *Ambystoma* salamanders (Greenwald, Gibbs, & Waite, 2009; Porej, Micacchion, & Hetherington, 2004), and here, data are presented from populations within the same forest patch and from populations at increasing distances away from this focal forest patch.

It was hypothesized that: (1) Genetic diversity would be lower (fewer alleles and higher inbreeding coefficients) in isolated populations, as compared to those in forest patches with more than one breeding pool. (2) Populations in separate forest patches would show genetic differentiation from one another, while those in the same patch would not. (3) Migration is not occurring between populations in separate forest patches that were historically connected by forested habitat and presumably gene flow.

## 2 METHODS

### 2.1 Study sites and field sampling

Salamanders were collected from eight breeding pools in five forest patches in rural Hardin County, Ohio (Figure 1). Three pools are within a 283‐ha forest (Lawrence Woods State Nature Preserve), and five additional pools are in separate forest patches at varying distances. Landscape measures determined using Google Earth (Google Inc. 2009) were forest patch area, pool area, distance between forest patches, and distance between pools (Table S1). For each sampled pool, the average distance to all other study pools and distance to nearest sample pool were recorded.

Adult *A. texanum* were captured in screen funnel traps placed in breeding pools in March 2009 and 2010. Tail tissue from up to 20 individuals was preserved in ethanol, and salamanders were released back into the pools. For sites without 20 adult samples, larvae were used so that we had a total of 20 individuals for each pool. *Ambystoma texanum* larvae were collected with dip nets during the summer of 2008.

### 2.2 DNA extraction and marker amplification

Genomic DNA was extracted and purified from tail tissue using standard proteinase‐K digestion and Wizard SV Genomic DNA Purification System (Promega). *Ambystoma texanum* larvae are difficult to distinguish from unisexual *Ambystoma* larvae (Brandon, 1961), which are common in some sample pools (Pfingsten & Downs, 1989). Larval species identification was confirmed using a molecular technique previously described (Rhoads, Krane, & Williams, 2010).

Eight polymorphic microsatellite loci were used as genetic markers in this study (Table S2); seven of the loci were characterized for *A. texanum* (Williams & Dewoody, 2004), and one locus was originally identified in *A. jeffersonianum* (Julian, King, & Savage, 2003) and found to cross‐amplify in *A. texanum* (J. P. Bogart, personal communication). The microsatellite loci were amplified by polymerase chain reaction, and fragment analysis of the products was carried out on an ABI 3100 and compared to a ROX 500 standard. PCR product size was determined using genescan v.3.1 and genotyper v.2.5 (Applied Biosystems).

### 2.3 Null alleles and relatedness

Each microsatellite locus was tested for conformance to Hardy–Weinberg equilibrium and for linkage disequilibrium using genepop v.4 (Rousset, 2008), and for the presence of null alleles and large allele dropout with micro‐checker v.2.2.3 (Van Oosterhout, Hutchinson, Willis, & Shipley, 2004). Several loci showed evidence of null alleles (Table S2) and were detected to have significant heterozygote deficiency in genepop. Genotypes were adjusted in micro‐checker according to Chakraborty, De Andrade, Daiger, and Budowle (1992).

To rebut the possibility that larval samples from the same pool were siblings, Queller and Goodnight's (1989) method of calculating pairwise relatedness (*r*_qg_) between individuals within populations was performed in genalex v.6.3 (Peakall & Smouse, 2006). Bootstrap resampling (1,000 bootstraps) provided 95% confidence intervals for *r*_qg_ for each population. A permutation test (999 permutations) for significant differences among mean population relatedness was performed, and 95% confidence intervals were derived for the expected range of *r*_qg_ under the null hypothesis of random mating. This method determines whether populations with larval samples showed higher within‐population relatedness than those with only adult samples, which would be expected if siblings had inadvertently been collected.

### 2.4 Within‐population diversity and population structure

genalex was used to determine population genetic measures for each population including mean number of alleles, number of private alleles, mean observed and expected heterozygosities, and inbreeding coefficients (*F*_IS_). Pairwise *F*_ST_ values were calculated in genalex for all population pairs with 999 permutations.

The program structure 2.3.1 (Pritchard, Stephens, & Donnelly, 2000) was used to assign individuals to inferred genetic clusters based on multilocus genotypes. Ten independent runs of *K* = 1–8 were performed with 10,000 Markov Chain Monte Carlo (MCMC) repetitions and a 10,000 burn‐in period assuming no admixture and independent allele frequencies and using sampling locations as priors. *K* was identified as the mean maximal value of ln *P*(*D*) and confirmed with calculation of Δ*K* (the second‐order rate of change between successive *K* values) (Evanno, Regnault, & Goudet, 2005). Once the most likely number of genetic clusters was identified, a final run of *K* was conducted with 10,000 MCMC repetitions and a 10,000 burn‐in period with the same parameter set.

In order to determine the amount of variation explained by the genetic clusters identified in structure, a hierarchal AMOVA (analysis of molecular variance) was performed (999 permutations) in genalex with populations assigned to regions based on the genetic clusters (region 1—Ha1, region 2—Ha2, region 3—all other sites). The *F*_ST_ analog Φ_ST_, which suppresses within‐individual variation, was used in order to detect subtle regional population structure.

### 2.5 Population size and migration rates

The Garza and Williamson (2001) method of calculating *M* was implemented to look for recent change in population size for sample populations. This method considers the number of alleles present in each population (for each locus) in relation to the expected number of alleles based on the difference between the largest and smallest allele size.

Maximum likelihood analysis was performed in migrate (Beerli, 2008) to estimate both migration rates and effective population sizes. The first run consisted of 10 short chains (50,000 sampled, 100 recorded) and one long chain (500,000 sampled, 5,000 recorded). Four‐chain adaptive heating at default temperatures (1, 1.5, 3, and 10,000) was used to increase efficiency. Two additional runs consisted of 10 short chains (10,000 sampled, 500 recorded) and three long chains (100,000 sampled, 5,000 recorded), combining the long chains and no adaptive heating.

Output from migrate gives ϴ values that can be converted to effective population size (*N*_e_) from the equation ϴ = 4*N*_e_μ. *N*_e_ was calculated assuming a microsatellite mutation rate (μ) of 5 × 10^−4^ (Garza & Williamson, 2001).

In addition to comparing bidirectional migration rates (*M*) for all population pairs, rates were averaged for groups of populations in two ways. First, pools that are in the same forest patch (Ha10 and Ha11, Lawrence Woods sites Ha3L1, 4, 5) were combined. All of the populations that comprise region 3 from the AMOVA (Ha3L1, 4, 5 and Ha7, Ha10, Ha11) were then combined in order to assess migration rates among the genetic clusters identified in structure.

### 2.6 Landscape genetics

A bivariate correlation matrix was created in pasw v.18 (2009) that included *F*_IS_ values, number of private alleles (*N*_P_), effective population sizes (*N*_e_), forest patch area, pool area, distance to nearest sample pool, and average distance to other sample pools. Several linear univariate and multiple regressions with *F*_IS_, *N*_P_, or *N*_e_ as the dependent variable and the landscape traits as independent variables were performed. Distance to nearest sample pool and average distance to other sample pools were significantly correlated (*p* < .001), so they were not included together in a multiple regression model. In genalex, a Mantel test (999 permutations) was used to test for a relationship between geographic and population genetic distances.

## 3 RESULTS

One hundred and sixty small‐mouthed salamanders were genotyped from eight breeding populations at eight highly polymorphic microsatellite loci (Table S2). A total of 143 alleles were detected, with the number of alleles per locus ranging from 12 (AjeD422) to 26 (Atex65 and Atex87). These results expand the size ranges for some Atex loci from those previously published (Williams & Dewoody, 2004) and are the first published for *A. texanum* from AjeD422.

### 3.1 Null alleles and relatedness

micro‐checker found no evidence of large allele dropout in our data set, but did detect the possibility of null alleles for six loci due to homozygous excess (Table S2). genepop corroborated in finding heterozygote deficiency for the same loci. The micro‐checker adjusted genotypes with added dummy alleles increased the observed heterozygosities so that they were very close to the original expected heterozygosities. Briefly, this method changes some homozygotes to heterozygotes by adding a dummy allele to some of the individuals in populations showing homozygous excess for a given locus. As explained in the Section 4, the data analysis was conducted with the original data set.

Increased relatedness (*r*_qg_) was not detected in populations with larval samples (Fig. S1). Three populations (Ha2, Ha3L1, Ha11) had significantly higher *r*_qg_ than expected under panmixia (*p* < .05), but two of those were comprised of mainly adult samples. Hardin 10, which was a mixture of adult and larval samples, had the only negative mean *r*_qg_ (−0.011).

### 3.2 Within‐population diversity

A summary of genetic results by population is presented in Table 1. The mean number of alleles per locus in each population ranged from 9.0 (Ha2) to 11.1 (Ha10). The overall mean observed heterozygosity was 0.543. Ha1 (0.488) and Ha2 (0.444) had the lowest H_O_, while Ha11 had the highest (0.624). In all populations, H_O_ was lower than expected, and inbreeding coefficients ranged from 0.201 (Ha11) to 0.461 (Ha2).

**Table 1:** Summary genetic statistics for sampled populations based on eight microsatellite loci

Population	*N*	A:L	*N*_A_	*N*_P_	*H*_O_	*H*_E_	*F*_IS_
Ha1	20.0	14:6	10.4	12	0.488	0.850	0.426
Ha2	20.0	18:2	9.0	2	0.444	0.789	0.461
Ha3L1	20.0	11:9	10.0	1	0.531	0.781	0.323
Ha3L4	20.0	15:5	10.5	2	0.600	0.803	0.265
Ha3L5	19.8	20:0	10.0	0	0.542	0.793	0.352
Ha7	19.9	19:1	10.3	4	0.596	0.781	0.242
Ha10	20.0	12:8	11.1	4	0.519	0.811	0.368
Ha11	19.9	20:0	10.4	1	0.624	0.775	0.201
Total	19.9	129:31	10.2	26	0.543	0.798	0.330

Mean number of individuals (*N*), adult‐to‐larvae ratio (A:L), mean number of alleles (*N*_A_), total number of private alleles (*N*_P_), mean observed heterozygosity (*H*_O_), mean expected heterozygosity (*H*_E_), and inbreeding coefficient (*F*_IS_).

### 3.3 Population structure

The greatest number of private alleles (12) was found in Ha1; all other populations had 4 (Ha7 and Ha10) or fewer (Table 1). Pairwise *F*_ST_ values indicate that Ha1 has highly significant genetic differentiation relative to all other sites (*p* = .001), and Ha2 has significant genetic differentiation from all sites (*p* ≤ .009) except Ha10 (*p* = .148), which is the closest geographically (Table 2). There is very little to no genetic differentiation among Ha7, Ha10, and Ha11, and between those sites and the Lawrence Woods sites (Ha3L1, 4, 5). There is, however, significant genetic differentiation among the Lawrence Woods sites.

**Table 2:** Pairwise population *F*_ST_ values^a^ (below diagonal) and geographic distances in km (above diagonal)

	Ha1	Ha2	Ha3L1	Ha3L4	Ha3L5	Ha7	Ha10	Ha11
Ha1	—	15.5	18.8	19.7	19.1	23.1	18.0	18.2
Ha2	0.065**	—	3.8	4.5	3.7	7.9	2.6	2.8
Ha3L1	0.047**	0.028**	—	1.0	1.3	4.4	1.6	1.3
Ha3L4	0.055**	0.040**	0.018*	—	0.9	3.5	2.0	1.6
Ha3L5	0.062**	0.015*	0.013*	0.018**	—	4.1	1.2	0.9
Ha7	0.064**	0.023**	0.007	0.001	0.003	—	5.3	5.1
Ha10	0.050**	0.005	0.000	0.005	0.000	0.000	—	0.3
Ha11	0.062**	0.020**	0.004	0.007	0.000	0.000	0.000	—

^a^ *p*‐Values based on 999 permutations.

**Figure 2:**
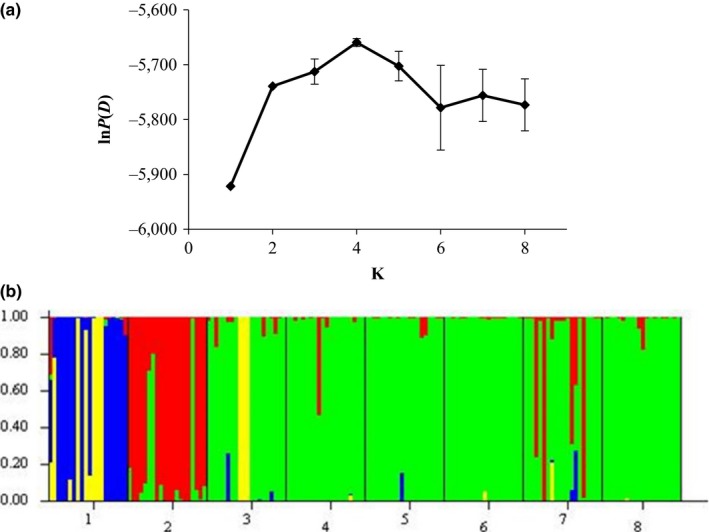
STRUCTURE results. (a) Mean values of ln *P*(*D*) from 10 independent runs for *K *=* *1–8. (b) Assignment of individuals to genetic clusters (*K* = 4) displayed by color. Each vertical line represents the probability that the individual belongs to a genetic cluster. Horizontal axis numbers represent sampled populations: 1‐Ha1, 2‐Ha2, 3‐Ha3L1, 4‐Ha3L4, 5‐Ha3L5, 6‐Ha7, 7‐Ha10, 8‐Ha11

**p = .001, *p < .05.

structure found that the most likely number of genetic clusters in the data is 4, which had the highest mean ln *P*(*D*) = −5659.65 (Figure 2a). Δ*K* was highest for *K* = 2 but showed a second peak at *K* = 4. The probability that each individual is from a particular genetic cluster is displayed in Figure 2b. Most individuals in Ha3L1, Ha3L4, Ha3L5, Ha7, Ha10, and Ha11 show membership to a single cluster. A second genetic cluster is comprised mainly of individuals from Ha2; a few individuals from Ha10 also may belong to this cluster. The last two genetic clusters are within Ha1.

The hierarchal AMOVA was performed with the populations assigned to three regions (Ha1, Ha2, all other sites). The AMOVA detected significant structure at the regional and within‐population scale, but not among the populations in region 3 (Table 3).

**Table 3:** Hierarchal AMOVA for three regions identified as genetic clusters in STRUCTURE^a^

Source of variation	*df*	SS	Variance	% of Variation
Among regions	2	56.3	0.58	6%
Among populations	5	47.0	0.02	0%
Within populations	152	1353.3	8.90	94%
Total	159	1456.6	9.51	100%

	Value	*p* b
Φ_RT_	0.061	.001
Φ_PR_	0.003	.243
Φ_PT_	0.063	.001

^a^Region 1—Ha1, region 2—Ha2, region 3—all other sites.

^b^Based on 999 permutations.

### 3.4 Population size

The effective population size (*N*_e_) estimates are shown in Table 4. Ha1 has the highest *N*_e_ = 600; all other sites had *N*_e_ estimates <500, with Ha2 having the lowest *N*_e_ = 429. Table 4 also shows the calculated mean *M* for each population, and all values are <0.71. Ha2 had the highest value (mean *M* = .707), while Ha1 had the lowest value (mean *M* = .499).

**Table 4:** Effective population size (*N*_e_) and mean *M* for sampled populations

	ϴ	*N*_e_	*M*	Var^a^
Ha1	1.20 (1.06–1.33)	600	0.678	0.025
Ha2	0.86 (0.80–0.94)	429	0.707	0.009
Ha3L1	0.99 (0.83–1.01)	497	0.539	0.052
Ha3L4	1.00 (0.93–1.07)	498	0.674	0.030
Ha3L5	0.98 (0.90–1.05)	488	0.654	0.040
Ha7	0.91 0.84–0.98)	456	0.545	0.044
Ha10	0.89 (0.84–0.96)	447	0.628	0.025
Ha11	0.89 (0.82–0.95)	443	0.612	0.016

ϴ values from maximum likelihood analysis in MIGRATE; 95% confidence intervals in parentheses.

^a^Var is the interlocus variance in *M*.

### 3.5 Migration rates

Bidirectional migration rates generated by migrate for all sampled populations are presented in Table 5. *M* ranged from 0.46 (Ha1 to Ha3L4) to 1.56 (Ha7 to Ha2); all rates were low in general. Given the size of the populations, *M* = 1 translates into an *N*_m_ = 0.25 (1 migrant every 4 generations). All seven rates <0.70 were coming to or from Ha1. The seven highest rates were >1.20 and involved Ha2, Ha7, Ha10, Ha11, and all sites from Lawrence Woods (Ha3L1, 4, 5).

**Table 5:** Bidirectional migration rates (*M*) inferring gene flow from maximum likelihood analysis in migrate

	Ha1	Ha2	Ha3L1	Ha3L4	Ha3L5	Ha7	Ha10	Ha11
Ha1		0.56 (0.45–0.69)	0.69 (0.57–0.85)	0.46 (0.37–0.57)	0.88 (0.73–1.04)	0.84 (0.71–1.03)	0.54 (0.43–0.65)	0.80 (0.67–0.97)
Ha2	0.73 (0.60–0.87)		1.03 (0.89–1.20)	0.94 (0.80–1.09)	1.10 (0.93–1.28)	0.97 (0.82–1.17)	1.41 (1.24–1.59)	1.14 (0.93–1.31)
Ha3L1	0.90 (0.76–1.09)	0.98 (0.83–1.18)		1.05 (0.90–1.21)	0.93 (0.77–1.10)	1.25 (1.08–1.44)	1.13 (0.98–1.29)	1.02 (0.87–1.18)
Ha3L4	0.71 (0.59–0.85)	1.17 (1.01–1.40)	1.17 (1.02–1.34)		1.01 (0.85–1.22)	1.08 (0.92–1.26)	0.96 (0.82–1.15)	0.99 (0.84–1.17)
Ha3L5	0.66 (0.54–0.83)	1.26 (1.04–1.45)	0.84 (0.71–0.99)	0.84 (0.71–1.02)		0.83 (0.70–0.99)	0.99 (0.83–1.14)	0.72 (0.59–0.88)
Ha7	0.51 (0.41–0.63)	1.56 (1.37–1.77)	1.17 (0.96–1.34)	1.07 (0.92–1.23)	0.81 (0.65–0.97)		0.90 (0.74–1.05)	0.93 (0.79–1.11)
Ha10	1.20 (1.04–1.42)	1.04 (0.88–1.21)	0.98 (0.83–1.14)	0.94 (0.80–1.12)	1.36 (1.15–1.56)	1.20 (1.00–1.38)		1.06 (0.91–1.26)
Ha11	0.62 (0.50–0.75)	1.21 (1.02–1.40)	1.13 (0.98–1.35)	1.23 (1.07–1.40)	1.17 (0.97–1.37)	0.86 (0.72–1.06)	0.85 (0.72–0.99)	

Direction of gene flow is from the population in the left column of the table to the population in the top row. 95% confidence intervals about the maximum likelihood value are in parentheses.

Comparing migration rates at the forest patch scale shows that all rates to and from Ha1 are ≤0.91 (Table 6). Rates to and from Ha2 are all ≥0.97, and rates to and from Ha7 are all ≥0.92 (both excluding Ha1). Ha 10,11 have migration rates ≥1.03 going to Ha2, Ha7, and the Ha3 sites; this patch has a rate coming from Ha2 of 1.28 and from Ha3 sites of 0.97. The Lawrence Woods sites (Ha3) have migration rates that range from 0.97 (to Ha 10,11) to 1.28 (from Ha2).

**Table 6:** Migration rates (*M*)^a^ from migrate between forest patches and between regions from AMOVA

	Region 1	Region 2	Region 3				
	Ha1	Ha2	Ha7	Ha10, 11	Ha3L1, 4, 5	Ha3, Ha7, 10, 11	Ha2, Ha3,7,10,11
Ha1	—	0.56	0.84	0.67 ± 0.18	0.68 ± 0.21	0.70 ± 0.17	0.68 ± 0.16
Ha2	0.73	—	0.97	1.28 ± 0.19	1.02 ± 0.08	1.09 ± 0.17	—
Ha7	0.51	1.56	—	0.92 ± 0.02	1.02 ± 0.19	—	—
Ha10, 11	0.91 ± 0.41	1.13 ± 0.12	1.03 ± 0.24	—	1.14 ± 0.16	—	—
Ha3L1, 4, 5	0.76 ± 0.13	1.14 ± 0.14	1.05 ± 0.21	0.97 ± 0.14	—	—	—
Ha3,Ha7,10,11	0.77 ± 0.25	1.20 ± 0.20	—	—	—	—	—
Ha2, Ha3, 7, 10, 11	0.76 ± 0.23	—	—	—	—	—	—

^a^Migration rates for patches with more than one pool show the average and standard deviation of *M* for all pools within that forest patch.

Confidence intervals for single pools in patches to other single pools given in Table 5.

Migration rates were also compared among the regions from the AMOVA (region1—Ha1, region 2—Ha2, region 3—all other sites) (Table 6). The migration rates between regions 1 and 2 (Ha1 to Ha2: 0.56, Ha2 to Ha1: 0.73) and between regions 1 and 3 (Ha 1 to region 3: 0.70, region 3 to Ha1: 0.77) were lower than between regions 2 and 3 (region 2 to region 3: 1.09, region 3 to region 2: 1.20).

### 3.6 Landscape genetics

**Figure 3:**
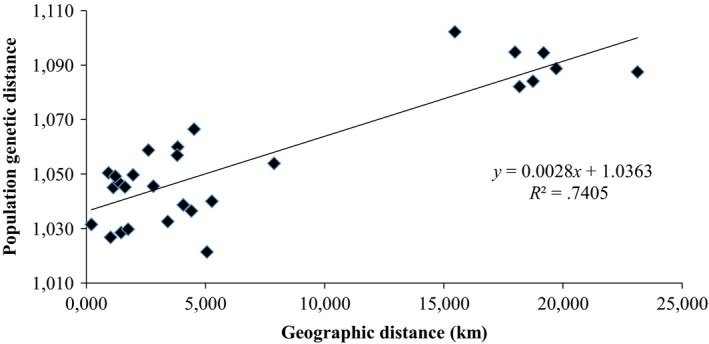
Isolation‐by‐distance analysis. Population genetic distance based on log‐transformed pairwise *F*_ST_ values. (Mantel test *r* = .861, *p* = .058)

The univariate regression analysis found no significant relationship between any of the landscape variables and *F*_IS_ values, or between number of private alleles and either forest patch area or pool area (Table 7A). Both average distance to other sample pools and distance to nearest sample pool were significantly correlated with the number of private alleles in a population (both *p* ≤ .001). Multiple regression models incorporating landscape variables were not significant for *F*_IS_ values. For number of private alleles in a population, models combining forest patch area and pool area with either average distance to other sample sites or distance to nearest sample site were significant but did not improve upon the univariate models with distance measures. The Mantel test found a positive relationship between geographic and population genetic distances, with geographic distance explaining 86.1% of the genetic differentiation among sampled populations (*r* ^2^ = .741, *p* = .058) (Table 7B, Figure 3).

**Table 7:** Landscape genetic analysis

Dependent variable	Independent variable	*r* ^2^	Significance^a^
(A)			
*N*_P_	Forest patch area	.270	ns
	Pool area	.154	ns
	Average distance	.907	***
	Distance nearest	.882	***
*F*_IS_	Forest patch area	.096	ns
	Pool area	.077	ns
	Average distance	.169	ns
	Distance nearest	.203	ns
*N*_e_	Forest patch area	.013	ns
	Pool area	.544	*
	Average distance	.673	*
	Distance nearest	.682	*
	Pool Area	.805	*
	Average distance		
	Pool area	.804	*
	Distance nearest		
(B)			
Population genetic distance	Geographic distance	.741	ns

(A) Linear regressions: number of private alleles (*N*_P_), inbreeding coefficient (*F*_IS_), effective population size (*N*_e_) (B) Mantel test.

^a^Significance ***p ≤ .001, **p ≤ .01, *p ≤ .05, ns (not significant).

The regression analysis using *N*_e_ as the dependent variable and landscape traits as independent variables found that pool area has a significant positive correlation with *N*_e_ (*r* ^2^ = .544, *p* = .037), but forest patch area does not (*r* ^2^ = .013, *p* = .788) (Table 7). Both average distance to other sample pools and distance to nearest sample pool were significantly correlated with *N*_e_ (*r* ^2^ = .673, *p* = .013 and *r* ^2^ = .682, *p* = .012, respectively). Pool area was combined with each distance measure into two separate multiple regression models, and both models showed improved fit (*r* ^2^ = .805, *p* = .017 and *r* ^2^ = .804, *p* = .017).

## 4 DISCUSSION

The populations in this study had low observed heterozygosities and high inbreeding coefficients. These results raise suspicion of the presence of null alleles in the microsatellite loci. Previous studies of *Ambystoma* population genetics have encountered the possibility of null alleles. Greenwald, Gibbs et al. (2009) found evidence for a null allele in two of nine microsatellite loci in *A. opacum*; genotypes were adjusted by adding a dummy allele (Chakraborty et al., 1992), and the adjusted genotype results were qualitatively no different from results with the original data set. Purrenhage et al. (2009) concluded that their *A. maculatum* data set contained missing data and that null alleles were not present at a frequency high enough to explain structure detected within ponds. Other researchers have employed three of the microsatellite loci that were amplified in this study, and their data show a range of observed heterozygosities in *A. texanum* populations (Gopurenko, Williams, & DeWoody, 2007; Williams & Dewoody, 2004; Williams, Gopurenko, Kemp, Williams, & DeWoody, 2009).

There are two possibilities that could explain the low observed heterozygosities in this data set: either there is a high frequency of null alleles in the loci or inbreeding has resulted in greater homozygosity than expected given the number of alleles present in the populations. It is expected that as a population is reduced in size, heterozygosity decreases more rapidly than the loss of alleles due to genetic drift (Hartl & Clark, 1997). Reproduction among close relatives results in an increase in homozygosity and can be detected by calculating the inbreeding coefficient for the population. The low frequency of potential homozygous null genotypes in our data set (no PCR product – see Table S2) suggests that null alleles are not present at a frequency high enough to be driving the low observed heterozygosities and high inbreeding coefficients. Furthermore, departure from Hardy–Weinberg equilibrium in six of the eight genetic markers suggests that the pattern of nonrandom mating occurs across loci, and not specifically in one or two loci, in which case null alleles would be a more plausible explanation.

It was hypothesized that breeding populations in forest patches with only one pool would show signs of decreased genetic diversity such as fewer alleles and lower observed heterozygosity. This was expected to be especially prominent in patches that appear isolated, specifically Ha1 and Ha2. It was found that all of the populations have fairly high inbreeding coefficients and the two most isolated populations (Ha1 and Ha2) had the highest *F*_IS_ values. Ha2 had both the highest *F*_IS_ and the lowest mean number of alleles.

The inbreeding coefficients for the populations within Lawrence Woods and the other forest patches (Ha10, 11 and Ha7) are smaller than Ha1 and Ha2, but still ≥0.201. The pools sampled in Lawrence Woods are all about 900 m from each other, which is a substantial distance for *A. texanum* to disperse, even within forest habitat. However, the presence of other unsampled pools within Lawrence Woods could serve as stepping stones that facilitate gene flow between our sites (Kimura & Weiss, 1964). This level of inbreeding in the Lawrence Woods sites may provide a baseline for population structure that is naturally present even in *Ambystoma* breeding populations that are in relatively close proximity to each other in forested habitat.

The mean observed heterozygosity was 0.543, which was very similar to the value found by Zamudio and Wieczorek (2007) of 0.534 for populations of *A. maculatum* in New York. Their study also detected higher than expected relatedness within ponds, although the range of *r*_qg_ (0.02 to 0.17) was higher than the *r*_qg_ values in this study (−0.011 to 0.040). The measure of relatedness is determined by comparing each population to the entire group of populations and therefore cannot be directly compared between studies. If a group of populations contains one or more populations with higher relatedness than the others, then they will have higher *r*_qg_ values and the range of *r*_qg_ values will be larger. The populations in this study may have relatedness measures that are more similar and therefore have *r*_qg_ values that are lower and within a smaller range.

Pairwise *F*_ST_ values of genetic differentiation between breeding populations are based on allelic variation and reflect the current population structure. It was hypothesized that populations in separate forest patches would show genetic differentiation, while those in the same forest would not. Ha1, which is 20 km distant from Lawrence Woods, is significantly different from all of the other sites. This degree of genetic difference was expected based on the low migration capability of this species. Ha2 is significantly different from all sites except Ha10, which is the site closest geographically. This may indicate that Ha2 has had more recent gene flow with Ha10 compared to the other populations. The *F*_ST_ values do reveal population structure within Lawrence Woods, but did not show significant differentiation between those sites and Ha10, Ha11, and Ha7, or among these latter three populations. The forest patch containing Ha 10 and 11 is separated from Lawrence Woods by a field that is about 250 m across, so migration between these two patches may be possible for *A. texanum*. The forest patch containing Ha7 is more than 1,800 meters from Lawrence Woods, and current salamander migration between these patches is unlikely.

It was hypothesized that migration is not occurring between populations in separate forest patches due to landscape change. One measure of a population's genetic isolation from other sampled populations is the presence of private alleles, which are assumed to be the result of mutation events. If there is very little or no gene flow between populations, then these new alleles are not shared between populations (Hartl & Clark, 1997). Ha1 had many more private alleles (12) than any other population. Ha7 and Ha10 both had four private alleles. Ha7 has the second highest average distance from other sample pools, so these private alleles suggest that gene flow from Ha7 to Lawrence Woods occurs at a very low rate or not at all. The presence of four private alleles in Ha10 was unexpected because Ha11 is within the same forest patch, and this patch is geographically close to Lawrence Woods. In the year prior to sampling, there had been development within this forest patch between the two pools (personal observation), but it is unlikely that the disruption of gene flow between them would be detected this quickly. There are a few private alleles in the Lawrence Woods pools, which supports the *F*_ST_ values in indicating population structure within this forest patch.

The identification of genetic clusters by structure largely corroborates the *F*_ST_ data and supports the idea of past gene flow among the Lawrence Woods sites and Ha7, 10, and 11. All of these sites are included in a single cluster that is separate from Ha1 and Ha2. The AMOVA found significant structure at the regional level (regions were based on the genetic clusters identified in structure), while no significant variation was explained at the population level for region 3. So while there may be some population structure among the Lawrence Woods sites, it is not great enough for structure to be able to assign individuals from those populations to separate genetic clusters. Ha2 is identified as a single cluster, but several individuals in its nearest population (Ha10) may belong to the Ha2 cluster. Ha2 and Ha10 also did not have a significant *F*_ST_ value. Ha1 is shown to contain two distinct genetic clusters, even although all individuals were sampled from the same pool. The *F*_ST_ values and number of private alleles show that Ha1 has highly significant genetic differentiation from the other sampled populations.

The program migrate provided estimates of historical gene flow and the opportunity to contrast migration rates prior to landscape change with current population structure detected with *F*_ST_ values and population assignment. It was hypothesized that all of the populations were historically connected by gene flow and that estimates of past migration rates would be similar among all sites, but the migrate results show that gene flow between Ha1 and all other sites was lower than among the rest of the populations. The genetic differentiation detected in this study between Ha1 and all other populations is probably not due to recent landscape change, but was present even historically. Ha1 may have been within a different metapopulation.

The estimates of past gene flow for all of the other populations suggest that they had higher migration rates with one another than any had with Ha1 and therefore may indicate that they were all within the same metapopulation historically. The migration rate estimates among the Lawrence Woods sites were not greater than those between the Lawrence Woods populations and Ha2 or Ha7. Although it would not be expected that any individual salamander ever dispersed from the Ha2 pool to Ha7, gene flow could have occurred indirectly among populations in the metapopulation following the stepping stone model prior to fragmentation between the forest patches that are left today (Kimura & Weiss, 1964).

Based on *F*_ST_ values and the assignment of individuals from Ha2 to a distinct genetic cluster, it appears that Ha2 is no longer connected by gene flow to the other sites. Deforestation and wetland loss may have disrupted gene flow between Ha2 and the forest patch containing Ha10 and Ha11. Migration today between the Lawrence Woods populations and Ha7 is unlikely because the forest patches are over 1,800 m apart and separated by cropland. However, differentiation was not detected between the Lawrence Woods populations and Ha7, as it was between the Lawrence Woods sites and Ha2. This may be because the Ha2 population is more isolated and smaller in size. Ha2's high inbreeding coefficient and lower mean number of alleles reflect a greater amount of nonrandom mating in this population and loss of alleles due to genetic drift. Ha7 is within a larger forest patch that also contains at least one other *A. texanum* breeding population (personal observation). The allelic diversity of Ha7 may be maintained by gene flow with this nearby unsampled population, so that Ha7's current allelic composition and diversity may remain similar to the Lawrence Woods populations even though migration may no longer occur between them. Four private alleles were found in Ha7, which supports recent lack of gene flow from this population to any of the others that were sampled.

Since migrate estimates bidirectional gene flow, it can be determined whether migration rates were symmetrical between populations or whether some populations were more often sources of migrants or metapopulation sinks. Overall, migration rates between the sampled populations were symmetrical, as the 95% confidence intervals around the maximum likelihood estimates overlap for most population pairs. Directionality was detected in two cases. Historically, Ha10 sent more migrants to both Ha3L5 and Ha11 than it received back from either. This appears to no longer be the case as four private alleles were detected in Ha10, suggesting dispersal of individuals from this population has been impaired. In addition, the rate of migration from Ha11 to Ha3L5 was greater than from Ha3L5 to Ha11. The migration rate estimates from Ha3L5 to the other sampled populations are generally lower than those from the other Lawrence Woods populations, Ha7, Ha10, Ha11, and Ha2.

Concerning Ha3L5, the highest rates of gene flow from Ha3L5 go to Ha2 and Ha10; these rates are higher than those between Ha3L5 and the other sampled populations in Lawrence Woods. This suggests that although Ha3L1, 4, 5 are together in a patch today, their past (and probably present) connectivity may be less than would be expected, as indicated by their *F*_ST_ values. The migration rates between Ha2, Ha10, and Ha3L5 are three of the highest estimates from all populations, and this may be due to their spatial arrangement and the intervening landscape configuration. The breeding pools for these three populations are directly adjacent to and on the north side of County Road 200, which runs east to west through southern Hardin County. These pools may have been historically connected by a natural drainage route that would have provided a moist dispersal corridor between them.

migrate also provided estimates of historical effective population sizes that may be most useful for comparisons between populations and are not necessarily accurate current population sizes. The range of effective population sizes calculated from the ϴ values generated by migrate is reasonable (Pfingsten & Downs, 1989). Ha1 had the largest *N*_e_ (600), while the other populations ranged from 429 (Ha2) to 498 (Ha3L4). Similar to the assignment of two genetic clusters to Ha1 in structure, migrate's larger estimate for population size is likely driven by within‐population structure.

Population size is important in determining whether an isolated population's genetic diversity is depressed by inbreeding and genetic drift. According to Garza and Williamson (2001), values of *M* (essentially the percentage of occupied allelic states) <0.70 indicate a recent reduction in population size. All of the sampled populations had mean *M* values below this threshold (except Ha2 with a mean *M* = 0.707). These low M values also indicate that the populations have lost alleles via genetic drift and suggest that the sampled populations have low connectivity to others. Migration among populations in a metapopulation should maintain allelic diversity by reintroducing alleles that have been lost. Ha1 had the lowest mean *M* (0.499) and the largest historical *N*_e_ estimate from migrate, which strongly suggests that this population was once larger in size and more genetically diverse than it is today.

Landscape factors that may limit population size for *Ambystoma* species include the size of the breeding pool and/or size of the forest patch. The regression analysis found that neither forest patch area nor pool area was correlated with inbreeding coefficients; however, effective population size did have a significant positive relationship with pool area—a result supported by other amphibian studies (Greenwald, Gibbs et al., 2009; Wang, Johnson, Johnson, & Shaffer, 2011). Overall, this suggests that while population size may be limited by breeding pool size, higher inbreeding coefficients are not directly related to the physical area of available breeding or terrestrial habitat. *F*_IS_ may be more influenced by the isolation of populations. Landscape change, such as habitat fragmentation that disrupts connectivity among populations, reduces genetic diversity in amphibians (Greenwald, Purrenhage, & Savage, 2009; Greenwald, Gibbs et al., 2009; Richardson, 2012).

In the regression analysis, effective population size was also significantly correlated with the two landscape distance measures, although the relationship was positive and not expected. This positive correlation was driven by Ha1 having the highest N_e_ and also being the most distant population from the others sampled. There was also a positive relationship between the landscape distance measures and inbreeding coefficients, but they were not significantly correlated. Lack of significance was probably driven by Ha7, which had one of the lowest F_IS_ values and is geographically distant from the other sites. The distance measures did, however, have a highly significant positive correlation with the number of private alleles in a population, which suggests that more isolated populations have less gene flow than among those closer together. This was also supported by the results of the Mantel test for isolation‐by‐distance that found that genetic distance between populations was positively related to their geographic distance (although it was not significant).

Zamudio and Wieczorek (2007) found that among *A. maculatum* populations sampled in New York, those populations ≥4.8 km from each other were genetically independent. This may suggest that metapopulations for *Ambystoma* species operate within this scale, as populations within this distance were more similar. The importance of this scale for amphibians is supported by a study on *Hyla arborea* (Arens et al., 2006), but a study on *A. maculatum* and *Rana sylvatica* in a landscape with abundant forest and wetland habitat found high connectivity up to 17 km (Coster, Babbitt, Cooper, & Kovach, 2015). The results of this study show that Ha2 is today genetically differentiated from Ha11 and the Lawrence Woods populations, which are all within 5 km. However, the historic migration rate estimates suggest that Ha 2 and Ha7 had gene flow at the same level that each had with the Lawrence Woods populations, even though Ha2 and Ha7 are almost 8 km apart.

## 5 CONCLUSIONS

This study of the population genetic structure of breeding populations in highly agricultural Hardin County, Ohio, was initiated in order to understand the impact of habitat destruction and fragmentation on *A. texanum*. All sampled populations exhibited high inbreeding coefficients. *Ambystoma* breeding populations may naturally have subtle population structure (Wang, Savage, & Shaffer, 2009); but in this study, those that were most isolated had the highest *F*_IS_ values (Ha1 and Ha2). Ha7 is geographically separated from the other populations sampled in this study by more than 1,800 m, but its relatively low *F*_IS_ value may be due to the presence of other breeding pools in the same forest patch that was not sampled. Ha7 is not an isolated population and demonstrates that genetic diversity is maintained in populations that are near other populations.

Ha2 may be an example of a small, isolated population. Estimates of historical gene flow suggest that this population was once connected by migration with the other populations, excluding Ha1. Pairwise *F*_ST_ values show that Ha2 has become genetically differentiated from the others, except Ha10. Ha2 had the highest inbreeding coefficient, the lowest mean number of alleles, and the smallest estimated *N*_e_. Ha1 also had a high inbreeding coefficient and is in a forest patch that contains only a single breeding pool. From aerial photographs and the past surveying of nearby forest patches and vernal pools, Ha1 appears to be an isolated population. The results of this study indicate that Ha1 has a larger population size than Ha2, which may explain why Ha1 had a higher average number of alleles.

The landscape traits of Ha1 and Ha2 typify forest patches in this region in general; sites such as Lawrence Woods (large forest size and multiple breeding pools) are rare to nonexistent in most counties. For this reason, *Ambystoma* breeding populations in many forest patches in this region may be experiencing inbreeding and genetic drift, especially if population sizes are small. Isolated populations lack the metapopulation structure that provides an avenue for movement of alleles, which enhances genetic diversity of populations and spreads adaptive alleles that are important for the persistence of a species in a changing environment. Numerous amphibian studies have detected reduced fitness in populations with decreased population genetic diversity (see Allentoft & O'Brien, 2010).

The estimates of historical gene flow in and around Lawrence Woods suggest that most of the populations sampled were once part of a metapopulation in a continuously forested habitat that had numerous pools with subtle population structure. Migration between populations in the metapopulation may have occurred at different rates between some of the populations, as data from this study suggest. Varying gene flow rates could have been influenced by geographic distance between populations and landscape traits, such as topography and soil moisture that impact vegetative composition. It cannot be assumed that breeding populations in the same forest patch today necessarily have high rates of gene flow between them. The Lawrence Woods sites illustrate this because there was greater genetic differentiation among those populations than between any of the Lawrence Woods sites and Ha7, Ha10, or Ha11. Conservation efforts should protect networks of breeding pools in close proximity, while considering natural dispersal routes and potential migration barriers, factors were not investigated in this study.

Landscape change, including deforestation and potentially loss of vernal pools, has disrupted the metapopulation structure detected to be present historically among our populations (excluding Ha1). Ha3L5 today is significantly differentiated from Ha2; but in the past, these two populations were connected by a level of gene flow higher than between Ha3L5 and the other Lawrence Woods populations we sampled, demonstrating that gene flow may have been highest between Lawrence woods pools and those that today are outside the forest. Today, the landscape between these two sites is agricultural and likely inhospitable to migrating amphibians. Even among the sampled sites without significant pairwise *F*_ST_ values (Ha10, 11, 7, and between those and Lawrence Woods), it is unlikely that gene flow between forest patches is occurring today because of both geographic distance and intervening land use, as supported by private alleles in Ha7 and Ha10. These populations may become genetically differentiated in the future, as mutations continue to introduce new alleles.

## CONFLICT OF INTEREST

None declared.

## AUTHOR CONTRIBUTIONS

E.R. and P.K.W. initiated and designed the project, and performed field sampling. E.R. performed the laboratory and data analyses, and wrote the first draft of the manuscript. C.K. advised on the laboratory and data analyses. All authors contributed to the final manuscript.

## DATA ACCESSIBILITY

Microsatellite genotypes will be submitted to Dryad for data archiving.
