# Supplementary material for: High inbreeding and low connectivity among Ambystoma texanum populations in fragmented Ohio forests
Source: Ecol Evol. 2017 Nov 15;7(24):11135–47. doi: 10.1002/ece3.3637 (PMC5743690; doi:10.1002/ece3.3637)
Supplement: Supplementary file 1 [file ECE3-7-11135-s001.docx]

**Figure S1**: Mean within population pairwise relatedness values (r_qg_). Error bars about the mean are 95% confidence intervals based on bootstrap resampling (1000 bootstraps). Red U and L bars represent upper and lower 95% confidence intervals for the null hypothesis of no difference across all populations (999 permutations). Three populations (*) show significantly higher r_qg_ than expected under random mating (p ≤ 0.05). Adult to larvae ratios (A:L) shown underneath population names out of a total of 20 samples per site.


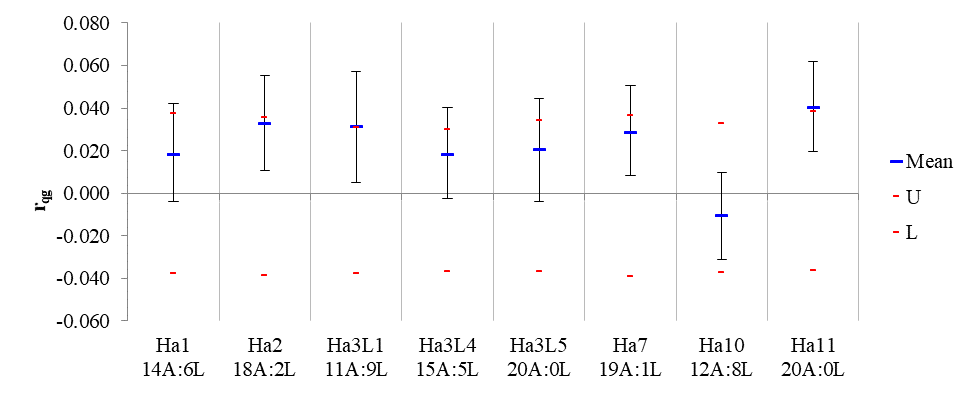


*

*

*

| **Table S1**: Sampled populations from Hardin County, Ohio | | | | | |  |
| --- | --- | --- | --- | --- | --- | --- |
| Population | Latitude | Longitude | Forest Area (ha) | Pool Area (m^2^) | Avg Dist (m)† | Dist Nearest (m)‡ |
| Ha1 | 40.5713 | -83.8438 | 9.51 | 3670 | 18915 | 15513 |
| Ha2 | 40.5531 | -83.6624 | 7.60 | 981 | 5831 | 2560 |
| Ha3L1 | 40.5687 | -83.6220 | 189 | 805 | 4600 | 1047 |
| Ha3L4 | 40.5634 | -83.6106 | 189 | 2513 | 4743 | 902 |
| Ha3L5 | 40.5569 | -83.6175 | 189 | 3662 | 4462 | 902 |
| Ha7 | 40.5636 | -83.5702 | 73.7 | 937 | 7624 | 3456 |
| Ha10 | 40.5562 | -83.6318 | 25.7 | 970 | 4450 | 344 |
| Ha11 | 40.5569 | -83.6295 | 25.7 | 967 | 4329 | 344 |
| †Average distance to all other sampled pools | | | |  |  |  |
| ‡Distance to nearest sampled pool | | |  |  |  |  |

| **Table S2**: Summary statistics for 8 tetranucleotide microsatellite loci across 8 sampled populations | | | | | | | | |  |
| --- | --- | --- | --- | --- | --- | --- | --- | --- | --- |
| **Locus** | **Total N** | **Mean N** | **Size (bp)** | **Total N_A_** | **Mean N_A_** | **Mean H_O_** | **Mean H_E_** | **Mean F** | |
| **Atex49*** | 160 | 20.0 | 96-175 | 16 | 10.6 | 0.706 | 0.857 | 0.176 | |
| **Atex65** | 159 | 19.9 | 258-390 | 26 | 16.5 | 0.812 | 0.915 | 0.117 | |
| **Atex74*** | 158 | 19.8 | 215-346 | 14 | 6.4 | 0.323 | 0.619 | 0.473 | |
| **Atex87*** | 160 | 20.0 | 113-288 | 26 | 12.4 | 0.394 | 0.885 | 0.553 | |
| **Atex89*** | 160 | 20.0 | 152-265 | 15 | 5.3 | 0.325 | 0.605 | 0.461 | |
| **Atex133*** | 160 | 20.0 | 151-208 | 15 | 10.0 | 0.438 | 0.838 | 0.476 | |
| **Atex141*** | 159 | 19.9 | 195-294 | 19 | 11.8 | 0.584 | 0.844 | 0.308 | |
| **AjeD422** | 160 | 20.0 | 192-257 | 12 | 8.8 | 0.763 | 0.821 | 0.074 | |
| **Total** |  | 19.9 |  | 143 | 10.2 | 0.543 | 0.798 | 0.330 | |
| Number of individuals (N), number of alleles (N_A_), observed heterozygosity (H_O_), expected heterozygosity (H_E_), and fixation index (F). *Microchecker detected homozygous excess. | | | | | | | | | |
